# Supplementary figures and images for: Comparison of Surface Properties of Sepiolite and Palygorskite: Surface Energy and Nanoroughness
Source: Nanomaterials (Basel). 2021 Jun 16;11(6):1579. doi: 10.3390/nano11061579 (PMC8235428; doi:10.3390/nano11061579)

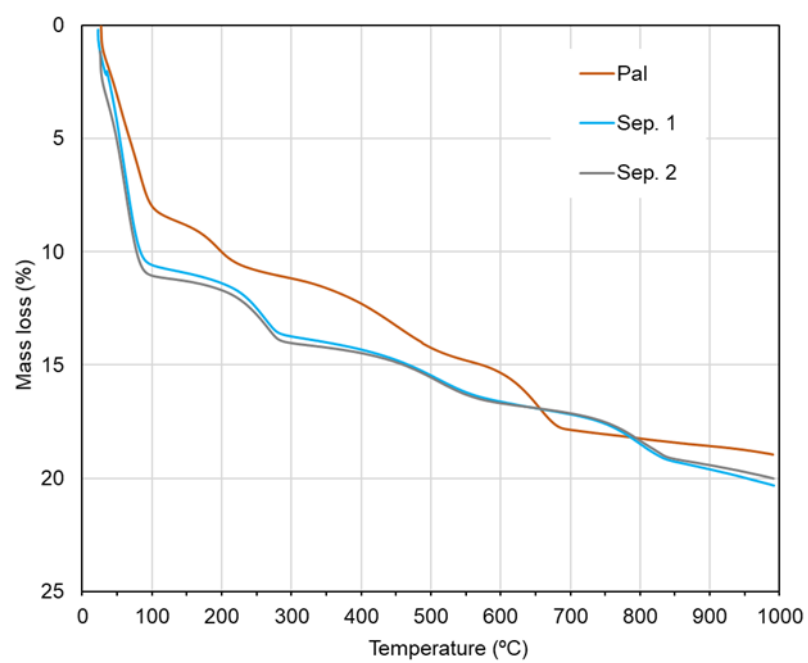

**Figure S1.** Thermogravimetry (mass loss) for the sepiolite and palygorskite samples.

Supplement: Supplementary file 1 [file nanomaterials-11-01579-s001.zip › nanomaterials-1195026-supplementary.pdf]
